# Supplementary material for: Sustained-input switches for transcription factors and microRNAs are central building blocks of eukaryotic gene circuits
Source: Genome Biol. 2013 Aug 23;14(8):R85. doi: 10.1186/gb-2013-14-8-r85 (PMC4054853; doi:10.1186/gb-2013-14-8-r85)
Supplement: Additional file 5 — HTML Browsable Motif Output. Zipped folder containing all WaRSwap and FANMOD motif output, viewable in a web browser. [file gb-2013-14-8-r85-S5.ZIP › HTML_browsable_motif_output/WaRSwap_ath_tair9/sigs_WSWRSLDraw_2-2000.pvals.heatmaps.html/motif_id_46_011101001_tftype_ath_upstream_-3000_0.html]

```
BG_MODEL = Rnew2
MOTIF_ID = 46_011101001
TF_TYPE = ath
UPSTREAM = -3000_0


PVals
FN_0.2	FN_0.4	FN_0.6	FN_0.8
dg_60.genes	0.079	0.049	0.013	0.75
dg_70.genes	0.084	0.037	0.011	0.733
dg_80.genes	0.07	0.048	0.007	0.727

ZScores
FN_0.2	FN_0.4	FN_0.6	FN_0.8
dg_60.genes	1.312	1.62	2.426	-0.921
dg_70.genes	1.294	1.718	2.535	-0.919
dg_80.genes	1.326	1.653	2.593	-0.906

StDevs
FN_0.2	FN_0.4	FN_0.6	FN_0.8
dg_60.genes	1078.363	626.853	145.805	15.093
dg_70.genes	1100.538	607.593	142.672	14.69
dg_80.genes	1062.949	645.03	141.476	14.671
```
